# Social Ecological Model of Problem Gambling: A Cross-National Survey Study of Young People in the United States, South Korea, Spain, and Finland

**DOI:** 10.3390/ijerph18063220

**Published:** 2021-03-20

**Authors:** Atte Oksanen, Anu Sirola, Iina Savolainen, Aki Koivula, Markus Kaakinen, Ilkka Vuorinen, Izabela Zych, Hye-Jin Paek

**Affiliations:** 1 Faculty of Social Sciences, Tampere University, 33014 Tampere, Finland; iina.savolainen@tuni.fi (I.S.); ilkka.vuorinen@tuni.fi (I.V.); 2 Department of Social Sciences and Philosophy, University of Jyväskylä, 40014 Jyväskylä, Finland; anu.r.s.sirola@jyu.fi; 3 Department of Social Research, University of Turku, 20500 Turku, Finland; akjeko@utu.fi; 4 Institute of Criminology and Legal Policy, University of Helsinki, 00014 Helsinki, Finland; markus.kaakinen@helsinki.fi; 5 Department of Psychology, University of Córdoba, 14004 Córdoba, Spain; izych@uco.es; 6 Department of Advertising and Public Relations, Hanyang University, Ansan 15588, Korea; hjpaek@gmail.com

**Keywords:** pathological gambling, social ecological model, adolescents, emerging adults, Internet, online communities, online casinos, consumer debt, advertising, impulsivity

## Abstract

Problem gambling among young people is an emerging trend globally. The online environment in particular offers various possibilities for gambling engagement. This is the first cross-national survey study using the social ecological model to analyze problem gambling, especially in the online context. The study aimed to analyze how different social ecological spheres explain problem gambling. Participants were young people aged 15–25 in the United States (*n* = 1212), South Korea (*n* = 1192), Spain (*n* = 1212), and Finland (*n* = 1200). The South Oaks Gambling Screen (SOGS) instrument measured problem gambling. The regression models analyzed problem gambling with measures of intrapersonal, interpersonal, organizational, and societal spheres. Spanish participants had the highest SOGS score for problem gambling. In all countries, the variations in problem gambling were best explained by the organizational sphere measures (26%) when compared to the intrapersonal (11%), interpersonal (5%), and societal (3%) spheres. In the full model, the organizational sphere measures had strong associations with problem gambling. These included consumer debt, online gambling community participation, online casino participation, and exposure to online pop-up advertisements. Problem gambling was also associated with conformity to group norms in the interpersonal sphere, and male gender and impulsivity in the intrapersonal sphere. Cross-national results were similar in different countries. Within the final model, gambling community participation had the strongest association with problem gambling (β = 0.23, *p* < 0.001). The online context plays a major role in problem gambling behavior. The social ecological model is a useful tool for tackling problem gambling and developing preventative measures.

## 1. Introduction

Problems caused by excessive gambling are a global concern [1,2,3]. Gambling behavior ranges from a harmless pastime activity to at-risk gambling that may develop into more serious forms of problem gambling and become pathological, thus creating negative psychological, social, and financial consequences for individuals, families, and society [4,5,6,7]. Currently, gambling and gaming increasingly take place online [8,9,10] and mainstream social media sites expose users to gambling content and activities [11,12,13]. Young people are the most active users of the Internet and social media sites and hence are at particular risk [14].

Due to the rapidly changing online environment, major gaps exist in research. Few studies have aimed to understand the behavioral and situational factors affecting gambling behavior and the development of gambling problems. This cross-national survey study used a social ecological model of gambling problems to analyze problem gambling among young people, aged 15 to 25.

### 1.1. Social Ecological Model for Gambling Problems

The need for understanding different types of human behavior social contexts is grounded in social psychology. Out of the classics of social psychology, Kurt Lewin, for example, postulated the classic equation *B* = *f*(*P*, *E*), noting that behavior is a function of a person and their environment [15]. This has been—and continues to serve as—a starting point for various social ecological models aimed at understanding human behavior from a holistic framework.

The most well-known example of a social ecological theory is Urie Bronfennerbrenner’s ecological systems theory of child development that analyzes wellbeing by using overlapping micro-, meso-, exo-, and macrosystems [16,17,18]. Later on, he also added a chronosystem reflecting time as a context [19]. Bronfenbrenner’s core idea is that human development takes place within these ecological systems. His theory and ideas have been widely applied in various social ecological models that share the idea that nested levels or spheres influence human behavior. The model generally has had a major influence on health promotion [20].

Bronfenbrenner’s work has also been applied in addiction research. The social ecological framework has been used in investigations of e-cigarette use [21], alcohol consumption [22,23], substance use [24,25,26], and high-risk sexual behavior [27], but less so in gambling. The only notable exception is a study using the Canadian Adolescent Gambling Inventory. The study used the social ecological model as a starting point but did not further assess the model’s usability [6]. Another research gap involves the Internet as a social ecological sphere. The social ecological framework has not been used in addiction research to understand the growing influence of the Internet on behavior. More work has been conducted in other fields, and the social ecological framework’s usefulness applied to the online context has been demonstrated in research on bullying [28].

Research on gambling problems has begun to search for comprehensive frameworks encompassing individual, social, and societal factors [29,30,31]. The Conceptual Framework of Harmful Gambling (CFHG) [29,30] emphasizes that gambling behavior and gambling harms are affected by general individual-level biological and psychological characteristics, but also by interpersonal relations and cultural factors. According to the CFHG, gambling problems are also shaped by the factors that determine the availability of different gambling environments, gambling types, gambling exposure, and resources available for harm reduction and problem gambling prevention within societies and communities.

Blaszczynski and Nower [4] noted ecological factors in the pathways model of problem and pathological gambling, but the theory only considers ecology as availability. However, addiction theories often simplify human behavior and neglect the the importance of social contexts in behavior. Some of them, such as Orford and West’s and others’ studies [32,33], comment on the relevance of societal and cultural contexts, but the core ideas are not social ecological. From a more social ecological point of view, addictions are formed only via settings that activate people to carry out certain activities despite their harmfulness [34]. 

**Figure 1:**
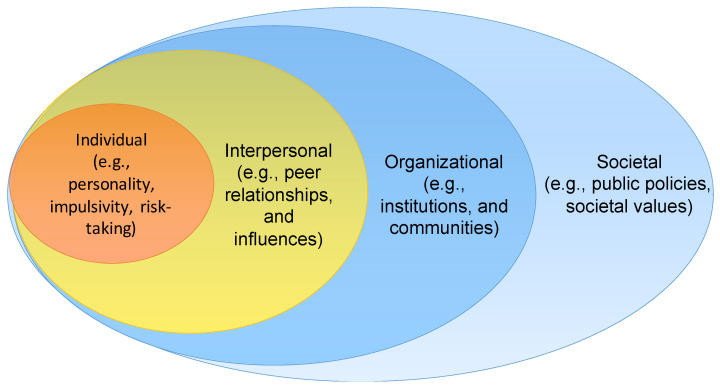
Social ecological model for investigating problem gambling behavior.

A social ecological model for problem gambling considers the development of gambling problems from the perspective of nested spheres, which combines both individual and situational factors. The spheres are grounded on the general ideas that the social ecological theory provides, indicating that human behavior and development take place in different contexts—from micro to macro—and depend on a person’s individual characteristics [18,19]. The intrapersonal sphere involves biopsychological factors such as age, gender, and personality. The interpersonal sphere also involves an individual’s interactions with others. The organizational (i.e., institutional) sphere includes potentially influencing factors available for individuals via social institutions. These meso-type factors include, for example, wider communities, institutional settings, and affordances. The societal sphere represents the macro level and includes public policies and cultural values. These four spheres are presented in Figure 1.

### 1.2. Evidence on Problem Gambling in Different Spheres

Research has well established the intrapersonal factors for gambling behavior. Males gamble more than females do in general, and gambling causes more problems for males than it does for females [1,35]. Problem gambling is more common among younger individuals [1,3]. Other intrapersonal variables related to personality are also important for understanding problem gambling. For example, impulsivity and sensation seeking are risk factors for problem gambling [35,36,37]. Similarly, some people are more likely to take risks, which can be associated with problem gambling [37]. Financial risk-taking is also common among young people [38,39]. Research also indicates that people with low self-esteem could be motivated to gamble [40]. 

Interpersonal factors include people’s social ties. In various studies, problem gamblers have reported lower perceived social support [41,42,43]. In adolescence and young adulthood, optimal relationships with family, peers, teachers, and classmates are important sources of support that protect individuals from problem gambling [44,45,46,47]. Although offline social ties provide a protective factor against problem gambling, online social ties involve a risk [47]. Furthermore, especially on social media, people easily become involved in various social cliques or bubbles [14,48] that could also pose a risk for problem gambling [49]. In addition, conformity to social norms influences gambling behavior [11,50].

Organizational factors include wider infrastructure, institutions, and communities that are not restricted to few individuals. Gambling venues such as online casinos are one example of such institutions. Access to casinos and other gambling content is fast and easy online. Online casinos in particular function both legally and illegally [2,10,51]. Furthermore, gambling communities often focus on sharing gambling tips. These types of communities were considered a risk factor for problem gambling in a previous Finnish study. The study noted that these communities, which young people visit online, most often focused on gambling activities and tips rather than the harm gambling causes or recovery from gambling problems [13].

Other organizational factors also influence gambling behavior. New types of opportunities for money lending and consumer credit, such as instant or payday loans, have been considered a major risk for financial difficulties, especially for young people [38,39]. These easy-access loans have also been identified as a problem in gambling research [52,53]. These loans occur via the Internet, and they are heavily marketed to users. Studies have also recognized the wide existence and ubiquity of online gambling marketing and advertising [54,55,56]. Moreover, problem gamblers receive more gambling advertisements, which likely increases their gambling [57,58]. Currently, online marketing offers more targeting and customization than ever, and problem gamblers likely receive advertisements about both gambling opportunities and instant loans on their social media feed. Problem gamblers often operate in a bubble [49], and escaping such a bubble is very difficult when marketing algorithms constantly target them with gambling advertisements.

The societal sphere is grounded in the idea of societies or broader unions (e.g., the EU) as macrosystems that influence people’s daily lives. This involves legislation in particular, but cultural and societal factors also play a role. For example, socioeconomic contexts, such as area-level income inequality and relative deprivation, contribute to youth problem gambling [44]. On the country level, expenditure on public health plays a role in youth problem gambling [45]. Globally, the prevalence of problem gambling ranges from 0% to 6% [1]. Figures also vary by definitions and measures used [59]. Cross-national studies indicate that the prevalence of problem gambling among young people varies between and within countries. For example, King and colleagues’ [60] review showed that the problem gambling rate among adolescents in the United States ranged from 1.7% to 33.8%, depending on the measure used [60]. Additionally, a recent ESPAD study showed that problem gambling among 16-year-olds ranged from 1.3% to 12% in European countries [61]. Given these types of inconsistencies in problem gambling research, it is important to use a cross-national approach when further analyzing the problem gambling phenomenon and its background factors. Moreover, cross-national research should go beyond simple comparisons of problem gambling prevalence rates, which are vulnerable to sampling and measuring differences.

### 1.3. This Study

This is the first cross-national survey study using a social ecological model to analyze problem gambling. Our study was grounded in a cross-national comparison between countries that are societally and culturally different: The United States, South Korea, Spain, and Finland. 

The United States is a liberal market-orientated country and South Korea is a technologically advanced East Asian country. Spain and Finland are part of the European Union but very different both culturally and societally. Finland represents a Nordic welfare state and Spain represents the South European regime [62,63]. Culturally, these countries also represent different areas on the Inglehart–Welzel world cultural map and are clearly distinct as societies [64,65]. Self-expression values are highest in Finland. Furthermore, Finland and South Korea score highly on secular-rational values in comparison to Spain and the United States in the cultural map [64,65]. The countries also differ in terms of collectivism, South Korea and Spain representing more collectivistic cultures than the United States and Finland [66,67]. The main similarity between our focus countries was that young people in all of these locations engaged actively on the Internet and social media [68].

In the United States, gambling is largely legalized and visible. Although federal legislations exist, each state holds its own laws and regulations for gambling, and the legal gambling age ranges from 18 to 21 in the country [69]. Although Finland and Spain are part of the EU, they regulate gambling nationally. Finland has a national monopoly system that the Lotteries Act regulates. Gambling services in Finland are provided solely by Veikkaus Oy, which is a state-owned company [70]. The legal gambling age limit in Finland is 18 years. In Spain, gambling is regulated on a national level by the Ministry of Consumption Gambling Act. The Directorate General for the Regulation of Gambling is the state-level authority that manages the regulations, licensing, and sanctioning of gambling activities. All gambling operators must attain a proper license for the operation, and individuals under the age of 18 are prohibited from gambling [71]. South Korea differs from the other three countries due to its significantly stricter gambling legislation. The National Gambling Commission Act regulates the gaming industry in South Korea, with the National Gambling Control Commission (NGCC) operating as its regulatory body. The NGCC controls the number of gambling businesses in the country as well as media content so that advertising is not released that may inspire excessive gambling. Limited gambling activities are legal and accessible for Koreans older than 18 years of age, as Korean citizens are only allowed to gamble in one of its casinos and participate in legal sports betting or lotteries [72,73].

Gambling is prevalent in all four countries. A review report found that the standardized problem gambling prevalence among the adult population was 1.5% in the United States (2001–2003), 1.5% in Finland (2011), 0.8% in South Korea (2011), and between 1.0% and 1.6% in different regions of Spain [73]. Problem gambling is visible even among young individuals. According to the ESPAD study, 3.9% of Finnish and 3.2% of Spanish 16-year-olds are problem gamblers [61]. National investigations in both countries also showed high figures. For example, in Finland, 5.3% of 18–24-year-olds reported problem gambling in 2019, according to the National Institute of Health and Welfare’s annual report [74]. Some recent reports from Spain have indicated relatively high figures of problem gambling as well. For instance, recent findings reported the rate of pathological gambling among 14- to 18-year-old youths is 7.6% for boys and 2% for girls [75]. However, samples, measures, and definitions vary from research to research [76,77,78]. In the U.S., a meta-analysis based on research on 13,000 college students estimated that 10.2% of them were probable pathological gamblers [79]. In a national sample from the United States, the prevalence of problem gambling was 5.4% among 18- to 30-year-olds [69]. A study conducted on South Korean college students found that, out of the sample used, 8.6% qualified as problem gamblers [80]. The results reflected the findings of previous prevalence research on problem gambling among South Korean college students with rates ranging from 11% to 14.6% [81]. In a national study, problem gambling prevalence was 3.4% for those aged 18 to 30 [82].

The country selection gave this study an excellent starting point because it enabled us to estimate the functionality of the social ecological model in different societal settings. The study focused on the online context and young people aged 15 to 25. Our starting point was to test the usability of a social ecological model for cross-national research and to analyze how well intrapersonal, interpersonal, organizational, and societal spheres explain problem gambling. Our hypotheses were grounded generally in the social ecological model of problem gambling and previous research on problem gambling; we expected that intrapersonal, interpersonal, and organizational factors would be associated with problem gambling. Exact variable-specific hypotheses were not preregistered due to the lack of previous studies using the social ecological model cross-nationally. Differences between countries were also investigated, but due to lack of previous research, we did not make any hypotheses for these differences.

## 2. Materials and Methods

### 2.1. Participants and Procedure

Our empirical evidence is based on cross-national survey data collected from 4816 young people aged 15 to 25 in Finland (*n* = 1200, 50.0% male), the United States (*n* = 1212, 49.83% female), South Korea (*n* = 1192, 49.58% male), and Spain (*n* = 1212, 51.24% male). The mean age of the participants was 21.29 (SD = 2.85) in Finland, 20.05 (SD = 3.19) in the United States, 20.61 (SD = 3.24) in South Korea, and 20.07 (SD = 3.16) in Spain. Of the participants, 58.85% were living with their parents (35.92%_FIN_, 51.16%_US_, 81.80%_SK_, 66.67%_SPA_), 61.00% were students (64.33%_FIN_, 53.96%_US_, 67.53%_SK_, 58.33%_SPA_), 22.57% had a university degree (13.42%_FIN_, 20.38%_US_, 28.10%_SK_, 28.38%_SPA_), and 5.38% were born abroad (4.08%_FIN_, 4.54%_US_, 0.59%_SK_, 12.21%_SPA_). See Table A1 in Appendix A for more information on the samples.

Data were collected from Finland in 2017, the United States in 2018, South Korea in 2018, and Spain in 2019 using identical YouGamble surveys focused on gambling, social media use, and wellbeing. Survey languages were Finnish in Finland, English in the United States, Korean in South Korea, and Spanish in Spain. The original YouGamble survey was in Finnish and professional-level translators converted it to English. Then, professional-level translators translated the Korean and Spanish surveys from the English version, and we used the back-translation process to confirm the translations’ accuracy.

Study participants were recruited using research panels the Survey Sampling International (currently Dynata) offers, which provides data solutions for research purposes globally. Dynata currently holds the largest research panels globally and it is available for different types of research. Such research panels typically have signed on participants who are contacted separately for each survey. Using research panels has become commonplace in social sciences and are considered a good alternative due to the difficulty of accessing hard-to-reach populations such as emerging adults [83]. The online data collection method avoids bias caused by traditional means such as phone surveys, especially when studying gambling problems [84].

The research group administrated all data collection and ran the survey using the Tampere University server. All four YouGamble surveys were collected using LimeSurvey software, and they were optimized for computers and mobile devices. Survey Sampling International sent a survey link to respondents. As our surveys were targeted to American, Finnish, South Korean, and Spanish young individuals in the 15–25 age group representing the general population, no additional inclusion criteria were applied. Sampling quotas were used to ensure the data matched the population of young people aged 15 to 25 in all the countries, especially in terms of age and gender, but also living area. Comparing country samples with the population showed only minor deviations; hence, analytical weights were not applied [52,68,84]. Besides these efforts to check and control the potential sources of bias, we ran additional data integrity checks during data collection on participant inclusion and participant effort [85,86]. Some of the full research project hypotheses were also preregistered [11]. Attention checks were run during the survey collection and the respondents who completed the survey excessively fast were not included in the data. We also looked separately for duplicate responses, and ran attention checks for response quality, matched item checks, and performed straight-lining checks. Additionally, we investigated the consistency of scales and sub-scales.

All respondents were volunteers and gave their consent for participation. In Finland, the legal age of consent is 15 years, so no parental consent was required, but it was still attained. Parental consent was also attained for the U.S., South Korean, and Spanish 15–17-year-old participants. All participants were informed about the study and were aware they could withdraw at any time without consequences. All responses were anonymous and no identifying information was collected. The ethics committee of the Tampere region in Finland reviewed the research protocol and stated the research did not include any ethical issues. The median survey response time was 14.54 min (15.30_FIN_, 14.49_US_, 12.32_SK_, 16.47_SPA_). The online survey format allowed questions to be mandatory and hence, no data are missing.

### 2.2. Measures

#### 2.2.1. Problem Gambling

Problem gambling was the outcome measure of our study, which we measured with the South Oaks Gambling Screen (SOGS). SOGS is among the most used measures for problem gambling [87,88]. It has also been used among 15- to 17-year-olds [89,90]. The scale had excellent inter-item reliability of 0.88, based on McDonald’s Ω: Ω_FIN_ = 0.89, Ω_US_ = 0.88, Ω_SK_ = 0.87, Ω_SPA_ = 0.86. SOGS reviews gambling activities from the past 12 months and scrutinizes factors indicating potential gambling problems based on 20 scoring items. SOGS scores’ range from 0 to 20, and higher scores indicate problem gambling. We used SOGS as a continuous measure in the analysis, but we also report results based on the cutoff of ≥8 for disordered gambling. A higher cutoff is considered better due to the potential for false positives with lower cutoffs [91].

#### 2.2.2. Intrapersonal Sphere

Besides gender and age measures of the intrapersonal sphere, this study included impulsivity, self-esteem, and risk-taking. 

Impulsivity was measured with the Eysenck Impulsivity Scale [36,92], with higher scores indicating higher impulsiveness. Response options were no (0) and yes (1) for all questions. The measure showed acceptable inter-item reliability: Ω = 0.69 (Ω_FIN_ = 0.75, Ω_US_ = 0.70, Ω_SK_ = 0.64, Ω_SPA_ = 0.67). In addition, the polychoric ordinal alpha coefficients for inter-item reliability of dichotomous scale were adequate: α_FIN_ = 0.87, α_US_ = 0.81, α_SK_ = 0.77 α_SPA_ = 0.80.

Self-esteem was measured with a single-item self-esteem scale [93]. Participants responded to the statement “I have high self-esteem” on a scale from 1 (*not very true of me*) to 10 (*very true of me*). 

Risk-taking was measured with a single-item statement (“I enjoy taking risks”), which was adapted from the 1979 National Longitudinal Survey of Youth (NLSY79) [94] and widely validated in various studies [95]. Response options ranged from 1 (*not very true of me*) to 10 (*very true of me*).

#### 2.2.3. Interpersonal Sphere

Interpersonal sphere measures included perceived social support, sense of belonging offline and online, involvement in social media identity bubbles, and conformity to group norms. These measures reflect individuals’ behavior in their close relationships and intimate groups.

Perceived social support was measured with a single item: “Do you feel that you receive support from your close ones when you need it?” The answer options were “never”, “sometimes”, and “often”. The options were categorized into a dummy variable indicating high social support (0 = *never* or *sometimes*, 1 = *often*). 

Offline belonging was measured with three items to indicate how strongly respondents felt they belonged to their close family members, friends, and school or work peers. All three items had response options ranging from 1 (*not at all*) to 10 (*very strongly*). The scale showed good inter-item reliability: Ω = 0.79 (Ω_FIN_ = 0.77, Ω_US_ = 0.83, Ω_SK_ = 0.82, Ω_SPA_ = 0.76). The scale was adjusted to a range of 1 to 10. A similar measure was used for online belonging. It asked how strongly participants felt they belonged to an online community [49]. Responses ranged from 1 (*not at all*) to 10 (*very strongly*). 

We used the six-item Identity Bubble Reinforcement Scale (IBRS-6) to measure involvement in social media identity bubbles (i.e., social cliques) [48]. The scale consisted of items such as “On social media, I belong to a community or communities that are important parts of my identity” rated from 1 (*does not describe me at all*) to 10 (*describes me completely*). The IBR-6 had high inter-item reliability: Ω = 0.88 (Ω_FIN_ = 0.79, Ω_US_ = 0.90, Ω_SK_ = 0.93, Ω_SPA_ = 0.86). The scale was adjusted to range from 1 to 10. 

Conformity to group norms was also included in the interpersonal sphere. This measure was based on an online experiment included in the middle of the survey [11]. The experiment simulated a social media setting and showed respondents gambling messages they could either like (thumbs up), dislike (thumbs down), or ignore. In the style of messages on social media, they were shown how other people had reacted to the same message. Numbers of likes and dislikes presented as reactions from other participants were manipulated in the experiment. Different gambling messages were shown four times. The scale ranged from 0 to 4, indicating the number of times respondents had agreed with the majority of others (i.e., selected the same response as about 85% of the other respondents). A higher score indicated higher conformity with the group norm. The scale had good inter-item reliability: Ω = 0.79 (Ω_FIN_ = 0.76, Ω_US_ = 0.78, Ω_SK_ = 0.82, Ω_SPA_ = 0.72).

#### 2.2.4. Organizational Sphere

Organizational sphere measures included consumer debt, participation in online casinos, participation in online gambling communities, and exposure to online pop-up gambling advertisements. These were related to institutions and considered wider than interpersonal factors.

Participants in all of the countries were asked whether they had taken payday loans or consumer debt in the past. In the Finnish survey, respondents were asked, “Have you ever taken instant loans, payday loans, or consumer credit?” Answer options were yes or no. These were categorized into a dummy variable: 0 = no consumer debt and 1 = consumer debt. In other surveys, respondents were asked whether they had taken a loan and then specified the type of loan taken: personal loans, consumer or credit card loans, cash advance loans, and payday loans were categorized as consumer debt. A dummy variable was created: 0 = no consumer debt, 1 = consumer debt. 

Online casino participation was measured with the question: “How often do you use online casino sites or other sites by gambling companies?” The answer options were “never”, “seldom”, “daily”, or “many times a day”. The answers were categorized into a dummy variable: 0 = no (never) and 1 = yes (at least seldom). 

Online gambling community participation was measured with the question: “How often do you use gambling-related discussion forums or communities?” The answer options were “never”, “seldom”, “daily”, or “many times a day”, and the response options were categorized into a dummy variable: 0 = no (never) and 1 = yes (at least seldom). Those who had participated in such communities were also asked a multiple-choice question on the content of such communities with the options of “gambling tips”, “users’ gambling experiences”, “gambling problems and recovery”, “gambling in general”, and “other issues”. The respondents were able to select multiple options.

Exposure to pop-up gambling advertisements was measured with the question: “Have you received online advertisements or announcements related to gambling (e.g., advertising messages from online casinos or pop-up windows)?” The answer options ranged from never to daily. Options were categorized into never, monthly (several times a month or less), or weekly (once a week or more often).

#### 2.2.5. Societal Sphere

The societal sphere in our cross-national investigation refers to the four countries’ societal spheres at the national level; hence, in this study, the societal sphere only reflects the macro level.

### 2.3. Statistical Modelling

Statistical analyses were run with Stata 16.1 software. The article reports descriptive results in Table 1 and the accompanying text. We used the χ^2^ test for the descriptive results. The main analyses focus on the regression models investigating how different social ecological spheres were associated with problem gambling in the four countries. We chose linear regression for the main method of estimating how well different spheres predicted gambling problems due to the comparability of results. We report standardized beta coefficients (*β*) that equal the correlation between the predictors and the outcome variable, which are comparable across models. In addition, coefficients of determination (R^2^) and *p* values for statistical significance are reported. Models are reported both separately for each country and by using aggregated data (*N* = 4816). Besides the regression coefficients, we also report partial eta squared (η^2^_p_) effect sizes in the text.

We did not detect problematic multicollinearity. The Breusch–Pagan test for heteroscedasticity showed some problems with heteroscedasticity of residuals, thus we ran the models using robust estimators of variance (i.e., sandwich estimator and Huber–White estimator). We ran additional checks for robustness because outliers were detected by looking at Cook’s distance measure, where values greater than 4/n may cause problems. We report the final model without outliers in Appendix B. In addition, country interactions were tested separately for each variable.

Appendix B also includes an alternative logistic regression model that uses a cutoff of ≥8 for problem gambling. We report odds ratios (ORs), their 95% confidence intervals, and *p* values for statistical significance. We also report a zero-inflated negative binomial (ZINB) regression model that takes into account overdispersion and excess zeroes of the outcome variable. ZINB is considered the most consistent model in these circumstances [96]. We ran ZINB models with robust estimation as the statistical literature suggests [97]. ZINB models isolate excess zeroes into a separate binary analysis and report remaining count variable analysis with incidence rate ratios (IRR) that are interpreted similarly to ORs in binary logistic regression (IRR > 1 indicates higher risk, and IRR < 1 indicates lower risk). We also report McFadden’s pseudo R^2^ coefficients, but these figures should be interpreted with caution as they are not comparable to linear regression coefficients.

## 3. Results

Descriptive statistics and information about the measures are reported in Table 1. SOGS score was highest among participants from Spain, followed by Finland, the United States, and South Korea. Based on a SOGS cut-off score of ≥ 8, 3.84% of the participants were disordered gamblers. The proportion of disordered gamblers was highest in Spain and lowest in South Korea (χ^2^ (3, *N* = 4816) = 33.56, *p* < 0.001).

Remarkable differences appeared, especially in the organizational sphere. For example, 42.33% of Finnish participants have visited online casino sites: 91% of them had been exposed to pop-up gambling advertisements, and 12.17% had taken on consumer debt. The figures in South Korea were very low, but participants from Spain and the United States also reported lower figures.

Online gambling community participation was highest among Spanish participants (25.58%), but also high among participants from Finland (14.42%) and the United States (13.94%), and low in South Korea (7.13%): χ^2^ (3, *N* = 4816) = 162.59, *p* < 0.001. These communities were generally about gambling advice, tips, and experiences from other users, and hence, could be considered pro-gambling communities. Only 20.62% of the participants had selected gambling problems or gambling problem recovery as topics of such communities.

Table 2 reports the findings of the linear OLS regression investigating the association of problem gambling and intrapersonal, interpersonal, organizational, and societal spheres. Results showed the organizational sphere (27%) and intrapersonal sphere (11%) best explained the variance of the SOGS score. Interpersonal (5%) and societal (3%) spheres were not as strong predictors. Notably, the different spheres’ models are very similar in all countries and only minor differences exist among them. All the significant effects have the same direction in all models. Robust predictors of problem gambling include, for example, male gender, impulsivity, risk-taking, conformity to group norms, and gambling community participation, which were statistically significant in all countries. 

Table 3 reports full models for all four countries and the complete aggregated dataset. Male gender, impulsivity, and risk-taking were statistically significant predictors of problem gambling in the intrapersonal sphere. None of the interpersonal sphere measures were significant in any of the countries, and effect sizes were small where significant. Out of the organizational sphere predictors, online gambling community participation was significant in all countries and in the aggregated model. Online casino participation was not significant in South Korea but remained significant in all other models even after adjusting for the number of factors.

Gambling community participation had the strongest correlation with gambling problems (β = 0.23). The age-, gender-, and country-adjusted effect size of online gambling community participation was large in all countries and in the aggregated model (η^2^_p_ = 0.20). Online casino participation also had relatively large effect sizes. In Finland, the association of online casino participation and problem gambling was strongest (β = 0.23, age- and gender-adjusted η^2^_p_ = 0.11). In addition, consumer debt was statistically significant in all the countries except the United States. Similarly, those who were exposed to pop-up gambling advertisements on a weekly basis reported higher problem gambling than others in all the countries except Finland. The full model that controlled all the spheres only showed a statistically significant difference between Spain and South Korea.

We ran robustness analyses first with linear regression by omitting outliers (see Table A2 in Appendix B). The model (*n* = 4546) explained 38% of the variance of problem gambling. The results were very consistent with the previous models, except some coefficients were higher, such as online gambling community participation (β = 0.28, *p* < 0.001). The robustness of our findings was further verified by running analyses with logistic regression and ZINB regression. These findings showed male gender, high impulsivity, and low self-esteem predicted problem gambling. In addition, consumer debt, online casino participation, online gambling community participation, and weekly exposure to pop-up gambling advertisements were associated with problem gambling. These findings generally underline the relevance of both the intrapersonal and organizational spheres in explaining problem gambling behavior.

Further country differences were analyzed with country interactions in the full linear model (*N* = 4816) with significant predictors. No differences occurred between women among the countries, but Spanish males had higher problem gambling than males in the United States (β = 0.05, *p* = 0.042) and South Korea (β = 0.06, *p* = 0.005). Impulsivity was not as strongly associated with problem gambling in South Korea as it was in Spain (β = −0.19, *p* = 0.001). Exposure to pop-up gambling advertisements was not associated with problem gambling in Finland as it was in other countries. This finding is also shown in Table 2 and Table 3, but the difference was also significant in the final model.

## 4. Discussion

This study adapted and developed a social ecological model for problem gambling and it is the first cross-national study to use a social ecological approach in analyzing problem gambling. This theoretical effort continues the search for more comprehensive frameworks in gambling research that also acknowledge social psychological and environmental factors [29,30,31]. We focused our article on the Internet as an emerging context for gambling among young people. We found that the social ecological model was useful in investigations of problem gambling, and our results were generally consistent across samples drawn from societies that are socially and culturally very different: the United States, South Korea, Spain, and Finland.

For the purposes of this study, different social ecological systems were analyzed as spheres, and we expected that intrapersonal, interpersonal, and organizational factors would be associated with problem gambling. Measures of organizational and intrapersonal spheres best explained the variance of problem gambling in all four countries. Significant and consistent predictors of problem gambling were online gambling community participation, male gender, and impulsivity. In addition, the interpersonal and societal spheres partly explained problem gambling. Conformity to group norms explained problem gambling in all countries.

Out of all measures the models included in the study, online gambling community participation had the strongest association with problem gambling. Within our study, these communities generally focused on supporting gambling activities, and not discussing potential gambling harm or overcoming gambling problems. Online gambling communities and their social norms hence promote harmful gambling ideas and are a major risk for problem gambling. This finding confirms the results gained in previous empirical research focused on Finland [13]. Other studies focused on gambling and gaming communities and their risks had similar findings, according to a systematic literature review [10]. Our results suggest that recovery-oriented online communities for ex-gamblers and people wishing to decrease or stop their gambling would be important. The benefits of online self-help groups and communities in overcoming problematic behaviors have been noted in other studies [98,99]; however, a challenge is how to make these kinds of communities attractive to young gamblers. Online communities should also be noted in the prevention and treatment of problem gambling, both as a potential risk factor for promoting and reinforcing problematic gambling, and a potential resource for recovery-oriented support.

The findings generally underline similarities across different countries, although problem gambling per se was less common in South Korea. The gender difference was bigger in Spain compared to other countries. The Spanish sample also had the highest rate of problem gambling. The role of impulsivity was less significant in South Korea and not statistically significant in the full model. Furthermore, exposure to pop-up gambling advertisements was not statistically significant among Finnish participants who also had very high exposure to such advertisements. Gambling advertising has been previously considered as aggressive in Finland, which might explain this finding [100]. Additionally, unlicensed gambling providers commonly advertise their services [101]. There were also some differences in country-specific results in the interpersonal sphere. Within the final model, the interpersonal sphere’s role was smaller. However, in Finland and South Korea, conformity to group norms was notably associated with problem gambling. However, the effect size was rather small. Despite these differences, our main result was that there were considerable similarities between countries, especially when investigating young people’s online behavior. Previous cross-national studies on youth online behavior have also noted that similar factors play a role in online risk factors, despite major cultural and societal differences [14].

Our results have practical implications. The results indicated several issues that could be regulated with policies, such as consumer debt, online casinos, and online advertisements. Payday loans and other short-term loans have been under serious discussion in countries such as Finland, where they pose a major risk for young people [38,52]. Online casinos have raised concern because they offer relatively easy access to gambling activities, in particular for younger people [9]. National strategies on regulating online casinos and gambling advertising differ significantly even within the EU. Bypassing restrictions and accessing offshore online casinos is also relatively easy [102]. Previous studies also call for new evidence and policies on regulating gambling advertising [56].

Within our study, we observed major differences in rates of young people accessing online casinos (e.g., Finland 42% vs. South Korea 8%). In South Korea, casino participation was rare compared to the other countries, and it was not a significant predictor of gambling problems. South Korea differs from other investigated countries with its stricter legislation, and hence, our results suggest strict laws might be an effective way to protect young people from gambling harm. Similarly, South Korean young people within our data saw fewer online pop-up gambling advertisements on a weekly basis (13%) than young people from Spain (38%), Finland (31%), and the United States (19%). These exposure rates, especially in Spain and Finland, are very high and indicate a need to find ways to regulate gambling marketing better.

Additionally, more attention should be paid to the online context and especially to online gambling communities. These communities could also be noted in gambling harm prevention. Other implications are related to better regulation of offshore casinos, illicit gambling advertising, and the availability of payday loans, but these are also related to national and international regulations. As the current online world sets quite a lot of challenges that are sometimes even difficult to tackle, it is crucial to find ways to support young people’s wellbeing. Our results also gave statistically significant results on the protective role of perceived social support and social belonging to significant others offline.

This study’s findings are limited to the four countries investigated with a cross-sectional design. The study is also limited by self-reported information that is potentially sensitive to social desirability bias when asking about problem gambling [103]. It is also likely that individuals with gambling problems are over-represented in online panels compared to the population estimates of problem gambling [87]. Longitudinal and experimental designs should be used in future studies to confirm our results. Moreover, the study’s measures were not designed solely for the purposes of the social ecological model, although we were able to use a wide range of measures to help understand personal, interpersonal, organizational, and societal factors. Future studies should continue using the social ecological model to examine problem gambling. They also should continue looking at contextual measures in different levels from the individual to societal. Measures on the actual gambling context could be especially useful. Future studies should also continue investigating the role of the online context and the emerging online risks in gambling behavior.

## 5. Conclusions

This cross-national study’s findings underline the benefits of the social ecological model in understanding and tackling problem gambling behavior. The results indicate the importance of the online digital infrastructure’s influence on gambling behavior. In our study, online gambling communities, online casinos, and pop-up gambling advertising, as well as fast access to financial resources such as payday loans, were associated with problem gambling. As a phenomenon, problem gambling is relatively similar in the countries representing Europe, North America, and Asia in this study. All countries have to tackle the emerging issues related to online gambling. The social ecological model would be useful in future studies on problem gambling.

## Figures and Tables

**Table 1:** Descriptive statistics of study variables.

		Finland	United States	South Korea	Spain	All
Dependent variable	Scale	M/%	M/%	M/%	M/%	M/%
Problem gambling (SOGS)	0–20	1.59	1.26	0.73	1.81	1.35
	≥8 points	3.67%	3.63%	1.76%	6.27%	3.84%
Independent variables						
Intrapersonal	Scale	M/%	M/%	M/%	M/%	M/%
Gender (male)	F/M	50.00%	49.83%	49.58%	51.24%	50.17%
Age	15–25	21.29	20.05	20.61	20.07	20.50
Impulsivity	0–5	1.96	1.90	1.56	2.05	1.87
Self-esteem	1–10	5.99	6.04	5.81	6.10	5.99
Risk-taking	1–10	5.12	5.74	4.21	5.41	5.12
Interpersonal	Scale	M/%	M/%	M/%	M/%	M/%
Perceived social support (high)	low/high	52.92%	41.34%	23.07%	48.76%	41.57%
Belonging offline	1–10	6.73	6.78	6.69	7.11	6.83
Belonging online	1–10	5.04	5.38	4.38	4.91	4.93
Social media identity bubble	1–10	4.63	5.96	5.26	5.75	5.40
Conformity to group norms	0–4	1.27	1.66	1.67	1.79	1.60
Organizational	Scale	%	%	%	%	%
Consumer debt	No/yes	12.17%	9.32%	5.54%	8.83%	8.97%
Online casino participation	No/yes	42.33%	18.23%	8.05%	28.22%	24.23%
Online gambling community participation	No/yes	14.42%	13.94%	7.13%	25.58%	15.30%
Pop-up gambling advertisements	Never	9.00%	27.15%	37.58%	8.17%	20.43%
	Max monthly	59.58%	53.80%	49.92%	53.71%	54.26%
	Weekly	31.42%	19.06%	12.5%	38.12%	25.31%

**Table 2:** Problem gambling explained by intrapersonal, interpersonal, organizational, and societal spheres in separate linear regression models.

	Finland		United States		South Korea		Spain		All	
Intrapersonal	β	*p*	β	*p*	β	*p*	β	*p*	β	*p*
Male gender	0.23	<0.001	0.14	<0.001	0.14	<0.001	0.22	<0.001	0.18	<0.001
Age	0.05	0.060	0.18	<0.001	−0.06	0.073	0.15	<0.001	0.09	<0.001
Impulsivity	0.19	<0.001	0.21	<0.001	0.12	<0.001	0.20	<0.001	0.19	<0.001
Self-esteem	−0.15	<0.001	0.03	0.383	−0.07	0.012	−0.05	0.097	−0.06	<0.001
Risk-taking	0.11	0.002	0.10	<0.001	0.19	<0.001	0.17	<0.001	0.16	<0.001
Model adjusted R^2^	12%		11%		8%		15%		11%	
Interpersonal	β	*p*	β	*p*	β	*p*	β	*p*	β	*p*
Perceived social support (high)	−0.08	0.012	−0.16	<0.001	−0.04	0.193	−0.20	<0.001	−0.09	<0.001
Belonging offline	−0.13	0.001	−0.03	0.418	−0.11	<0.001	−0.03	0.399	−0.08	<0.001
Belonging online	0.04	0.149	0.10	0.001	0.13	<0.001	0.16	<0.001	0.13	<0.001
Social media identity bubble	0.02	0.630	0.08	0.008	0.08	0.002	0.12	<0.001	0.07	<0.001
Conformity to group norm	0.14	<0.001	0.10	<0.001	0.08	0.002	0.06	0.014	0.08	<0.001
Model adjusted R^2^	5%		6%		4%		11%		5%	
Organizational	β	*p*	β	*p*	β	*p*	β	*p*	β	*p*
Consumer debt	0.19	<0.001	0.06	00.067	0.18	<0.001	0.10	0.004	0.12	<0.001
Online casino participation	0.22	<0.001	0.17	0.002	0.12	0.175	0.22	<0.001	0.20	<0.001
Online gambling community partic.	0.25	<0.001	0.26	<0.001	0.33	0.001	0.26	<0.001	0.28	<0.001
Pop-up gambling advertisements (ref. never)										
Max monthly	−0.04	0.504	0.07	0.001	0.05	0.005	0.06	0.018	0.05	<0.001
Weekly	−0.03	0.650	0.17	<0.001	0.11	0.001	0.18	<0.001	0.13	<0.001
Model adjusted R^2^	22%		23%		29%		26%		26%	
Societal									β	*p*
Country difference (ref. Spain) Finland	-	-	-	-	-	-	-	-	−0.04	0.049
United States	-	-	-	-	-	-	-	-	−0.09	0.000
South Korea	-	-	-	-	-	-	-	-	−0.18	0.000
Model adjusted R^2^									3%	

**Table 3:** Problem gambling explained by the full social ecological model in linear regression models.

	Finland		United States		South Korea		Spain		All	
Intrapersonal	β	*p*	β	*p*	β	*p*	β	*p*	β	*p*
Male gender	0.12	<0.001	0.08	0.001	0.08	<0.001	0.13	<0.001	0.11	<0.001
Age	−0.06	0.015	0.10	0.001	−0.08	0.009	0.06	0.026	0.01	0.398
Impulsivity	0.13	<0.001	0.14	<0.001	0.04	0.102	0.13	<0.001	0.12	<0.001
Self-esteem	−0.06	0.027	0.01	0.867	−0.06	0.028	−0.03	0.289	−0.03	0.048
Risk-taking	0.05	0.094	0.05	0.092	0.07	0.010	0.07	0.003	0.07	<0.001
Interpersonal										
Perceived social support (high)	−0.03	0.206	−0.06	0.053	0.02	0.490	−0.09	0.003	−0.06	<0.001
Belonging offline	−0.07	0.029	−0.01	0.864	−0.04	0.236	−0.02	0.596	−0.04	0.030
Belonging online	−0.02	0.411	0.02	0.494	0.00	0.908	0.08	0.003	0.03	0.033
Social media identity bubble	0.02	0.446	0.00	0.946	0.03	0.158	0.02	0.368	0.03	0.058
Conformity to group norm	0.06	0.037	0.04	0.089	0.06	0.002	0.02	0.435	0.04	<0.001
Organizational										
Consumer debt	0.16	<0.001	0.03	0.352	0.18	<0.001	0.07	0.034	0.11	<0.001
Online casino participation	0.22	<0.001	0.14	0.011	0.11	0.214	0.16	<0.001	0.17	<0.001
Online gambling comm. partic.	0.20	<0.001	0.23	<0.001	0.31	0.002	0.21	<0.001	0.23	<0.001
Pop-up gambling advertisements (ref. never)										
Max monthly	−0.02	0.739	0.04	0.045	0.03	0.106	0.04	0.205	0.02	0.073
Weekly	−0.02	0.790	0.13	<0.001	0.09	0.008	0.13	<0.001	0.09	<0.001
Societal										
Country difference (ref. Spain) Finland	-	-	-	-	-	-	-	-	−0.01	0.446
United States	-	-	-	-	-	-	-	-	−0.03	0.056
South Korea	-	-	-	-	-	-	-	-	−0.05	0.004
Model adjusted R^2^	28%		27%		31%		33%		31%	

## Data Availability

YouGamble 2017–Finnish Data are publicly available in the Finnish Social Science Data Archive (http://urn.fi/urn:nbn:fi:fsd:T-FSD3399) (accessed on 19 March 2021). Data from the United States, South Korea, and Spain will be made publicly available in the Finnish Social Science Data Archive during 2021. The data are available from the corresponding author (A.O.) with a reasonable request.

## Appendix A

**Table A1:** Descriptive statistics of the study participants.

	Finland	United States	South Korea	Spain
Age (mean)	21.29	20.05	20.61	20.07
Male (%)	50.00	49.83	49.58	51.24
University degree (%)	13.42	20.38	28.1	28.38
Occupational status				
Student (%)	64.33	53.96	67.53	58.33
Working (%)	20.33	34.16	21.82	31.36
Unemployed/other (%)	15.34	11.88	10.65	10.31
Born abroad (%)	4.08	4.54	0.59	12.21
Lives with parents (%)	35.92	51.16	81.80	66.67
Significant financial support from parents or relatives (%) *	17.56	35.47	65.90	58.42

Note. * this question was asked only those not living with parents. The exact question was: “how significant of a portion of your average monthly income is financial support provided by your parents or other relatives?”.

## Appendix B

**Table A2:** Alternative linear, logistic, and zero-inflated negative binomial regression models predicting gambling problems.

	Linear		Logistic				ZINB			
	β	*p*	OR	95%	CI	*p*	IRR	95%	CI	*p*
Intrapersonal										
Male gender	0.11	<0.001	1.96	1.36	2.82	<0.001	1.30	1.20	1.40	<0.001
Age	0.02	0.145	1.01	0.95	1.07	0.725	0.99	0.97	1.00	0.039
Impulsivity	0.10	<0.001	1.33	1.19	1.48	<0.001	1.08	1.06	1.11	<0.001
Self-esteem	−0.03	0.042	0.90	0.82	0.99	0.030	0.97	0.95	0.99	0.005
Risk-taking	0.08	<0.001	1.07	0.99	1.17	0.099	1.03	1.01	1.04	0.007
Interpersonal										
Perceived social support (high)	−0.07	<0.001	0.70	0.47	1.04	0.076	0.90	0.82	0.98	0.018
Belonging offline	−0.02	0.178	0.91	0.81	1.01	0.080	0.96	0.94	0.99	0.004
Belonging online	0.03	0.052	1.08	0.99	1.17	0.068	1.02	1.00	1.04	0.013
Social media identity bubble	0.03	0.029	1.13	1.01	1.26	0.035	1.02	1.00	1.05	0.074
Conformity to group norm	0.04	<0.001	1.08	0.92	1.27	0.349	1.04	1.01	1.08	0.016
Organizational										
Consumer debt	0.11	<0.001	2.91	2.00	4.23	<0.001	1.23	1.11	1.36	<0.001
Online casino participation	0.22	<0.001	2.56	1.58	4.14	<0.001	1.20	1.09	1.32	<0.001
Online gambling comm. partic.	0.28	<0.001	2.68	1.70	4.20	<0.001	1.39	1.26	1.54	<0.001
Pop-up gambling adv. (ref. never)										
Max monthly	0.03	0.014	1.39	0.67	2.90	0.381	1.02	0.88	1.19	0.748
Weekly	0.09	<0.001	2.41	1.13	5.14	0.022	1.17	1.00	1.36	0.047
Societal										
Country difference (ref. Spain) Finland	0.00	0.929	0.76	0.48	1.20	0.237	1.06	0.94	1.18	0.348
The U.S.	−0.05	0.003	0.80	0.52	1.22	0.295	0.90	0.79	1.03	0.135
South Korea	−0.06	<0.001	0.66	0.38	1.13	0.132	1.07	0.97	1.18	0.195
Model N	4546 *		4816				4816			
Adjusted R^2^	38%									
Pseudo adj. R^2^ (McFadden)			24%				42%			

Note. *** Outliers omitted from the linear regression model.
